# Hamiltonian Paths Through Two- and Three-Dimensional Grids

**DOI:** 10.6028/jres.110.012

**Published:** 2005-04-01

**Authors:** William F. Mitchell

**Affiliations:** National Institute of Standards and Technology, Gaithersburg, MD 20899-8910

**Keywords:** adaptive grid refinement, Hamiltonian path, load balancing, refinement-tree partition

## Abstract

This paper addresses the existence of Hamiltonian paths and cycles in two-dimensional grids consisting of triangles or quadrilaterals, and three-dimensional grids consisting of tetrahedra or hexahedra. The paths and cycles may be constrained to pass from one element to the next through an edge, through a vertex, or be unconstrained and pass through either. It was previously known that an unconstrained Hamiltonian path exists in a triangular grid under very mild conditions, and that there are triangular grids for which there is no through-edge Hamiltonian path. In this paper we prove that a through-vertex Hamiltonian cycle exists in any triangular or tetrahedral grid under very mild conditions, and that there exist quadrilateral and hexahedral grids for which no unconstrained Hamiltonian path exists. The existence proofs are constructive, and lead to an efficient algorithm for finding a through-vertex Hamiltonian cycle.

## 1. Introduction

Adaptive grid refinement has been shown to be an effective tool for reducing the size of the grid (and consequently the linear system) required for a given accuracy when numerically solving partial differential equations. Problems involving singularities or multi-scale behavior practically require adaptive refinement. When implemented for parallel computers, dynamic load balancing is required to keep all of the processors busy. This involves partitioning the grid into equal sized pieces (in some measure), and distributing the data among the processors accordingly. Many of the methods for computing a partition are based on a linearization of the elements (or path through the elements) in a two-dimensional or three-dimensional grid, and then cutting the linear sequence into pieces of equal size. Some of the methods that fall into this category are the space filling curves [1], OCTREE [2], and refinement-tree [3] methods. Further information on partitioning methods can be found in Ref. [4].

The space filling curve and OCTREE methods are not guaranteed to create connected partitions, which may be a desirable property. The refinement-tree method is guaranteed to give connected partitions provided that an appropriate linearization of the initial coarse grid is given. Such a linearization would be a Hamiltonian path, i.e., a path which passes from an element to a neighboring element and goes through each element exactly once. Heber et al. [5] proved that, for grids consisting of triangles, a Hamiltonian path always exists under very mild conditions. Moreover, the proof is a constructive proof which leads to an efficient algorithm to find a Hamiltonian path. However, the refinement-tree partitioning algorithm requires a stronger result in which the Hamiltonian path always passes through vertices when moving from one element to the next (as opposed to passing through edges), and does not go out the same vertex through which it came in. We call this a through-vertex Hamiltonian path. Heber’s algorithm produces Hamiltonian paths that pass through both vertices and edges. Additionally, Ref. [5] only addresses triangles, not the other elements considered in this paper.

The main result of this paper is the proof that there always exists a through-vertex Hamiltonian path in grids consisting of triangles or tetrahedra, under very mild conditions. The proof is constructive, which leads to an algorithm to construct such a path. We do not explicitly give the algorithm in this paper, but it follows easily from the proofs of the main theorems. Little is known about the conditions under which a Hamiltonian path exists in grids consisting of quadrilaterals or hexahedra. An algorithm is given that might find a through-vertex Hamiltonian path in a quadrilateral or hexahedral grid, if one exists, and is likely to give a broken path with a small number of discontinuities, i.e., something close to a through-vertex Hamiltonian path.

The remainder of the paper is organized as follows. In Sec. 2 we introduce the notation and define the terms used in this paper. Section 3 addresses triangles and tetrahedra. It reviews previously known results and presents the main results of this paper. In Sec. 4 we discuss quadrilaterals and hexahedra. Section 5 contains the conclusions.

## 2. Definitions

For the purposes of this paper, an *element, E*, is a triangle, quadrilateral, tetrahedron, or hexahedron. An element contains *vertices, v*, and *edges, e*, with the obvious definitions, and three-dimensional elements contain *faces, f*.

A *grid, G*, is the union of a collection of elements, {*E_i_*}, all of the same kind, such that *G* = ∪*E_i_* is a connected, bounded region in ℜ^2^ or ℜ^3^, and $\overset{\circ }{E_{i}}\cap \overset{\circ }{E_{j}}=\phi$, *i* ≠ *j*, where $\overset{\circ }{E}$ denotes the interior of element *E*, and *ϕ* is the empty set. We say that *E_i_* is an element of *G*, *E_i_* ∈ *G*. A vertex of *E_i_* is a *boundary vertex* if it lies on the boundary of *G*, and an *interior vertex* if it is not a boundary vertex. We say the size of *G*, |*G*|, is *N* if there are *N* elements in *G*. A *triangular grid, quadrilateral grid, tetrahedral grid*, and *hexahedral grid* is a grid consisting entirely of triangles, quadrilaterals, tetrahedra and hexahedra, respectively.

**Fig. 1:**
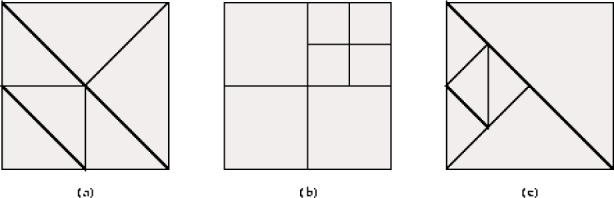
(a) A conforming triangular grid. (b) A 1-nonconforming quadrilateral grid. (c) A grid that is neither conforming nor 1-nonconforming, because an element edge contains more than one hanging node.

A grid is said to be conforming if *E_i_* ∩ *E_j_*, *i* ≠ *j*, is a common vertex, common edge, common face or empty. A vertex of an element is called a *hanging node* if it lies in the interior of an edge or face of another element. It follows immediately from the definition that a conforming grid has no hanging nodes. A grid is said to be *1-nonconforming* if there is at least one hanging node in the grid, all edges contain at most one hanging node, the interior of all faces contain at most one hanging node, and the intersection of two elements is a vertex, edge or face of one of the elements, or empty. See Fig. 1 for examples of conforming and 1-nonconforming grids. This paper will primarily consider triangular and tetrahedral grids that are conforming, and quadrilateral and hexahedral grids that are conforming or 1-nonconforming.

A *path* with *length n* in a grid *G* is a sequence of elements, *E*_1_*E*_2_…*E_n_*, *E_i_* ∈ *G*, *i* = 1, *n*, with *E_i_* ∩ *E_i_*_+ 1_ ≠ *ϕ*, and *E_i_* ≠ *E_i_*_+ 1_, *i* = 1, *n* − 1. A *cycle* of length *n* is a path of length *n* + 1 in which *E*_1_ = *E_n_*_+ 1_. A *Hamiltonian path* is a path in which every element in *G* appears exactly once. A *Hamiltonian cycle* is a cycle in which every element in *G* appears exactly once except for *E*_1_ = *E_n_*_+ 1_, which appears exactly twice.

A sequence of elements *E*_1_*E*_2_…*E_n_* for which there exists an *i* with *E_i_* ∩ *E_i_*_+ 1_ = *ϕ* is called a *broken path*, and the sequence *E_i_ E_i_*_+ 1_ is called a *discontinuity* in the path.

A *through-vertex path* (*through-vertex cycle*) is a path (cycle) in which the passage from one element to the next is specified as a common vertex, and the path does not pass through the same vertex when entering and exiting an element. Specifically, it is *E*_1_*v*_1_*E*_2_*v*_2_…*v_n_*_−1_ *En* where *v_i_* ⊆ *E_i_* ∩ *E_i_*_+ 1_, *i* = 1, *n* − 1, *v_i_* ≠ *v_i_*_+ 1_, *i* = 1, *n* − 2, and *E*_1_*E*_2_…*E_n_* is a path (cycle). We say that the path *passes through v_i_* when going from *E_i_* to *E_i_*_+ 1_, and that we come into *E_i_* through the *in-vertex v_i_*_− 1_ and leave *E_i_* through the *out-vertex v_i_*. A *through-edge* path and cycle, and *through-face* path and cycle are defined similarly. A *through-vertex Hamiltonian path* is a through-vertex path that is also a Hamiltonian path, and the definitions of the obvious similar terms are similar. An *unconstrained path* is one that may pass through any of vertices, edges or faces. Although the term is redundant, we will use it when we want to emphasize that the path is not constrained to pass through only vertices, edges or faces.

**Fig. 2:**
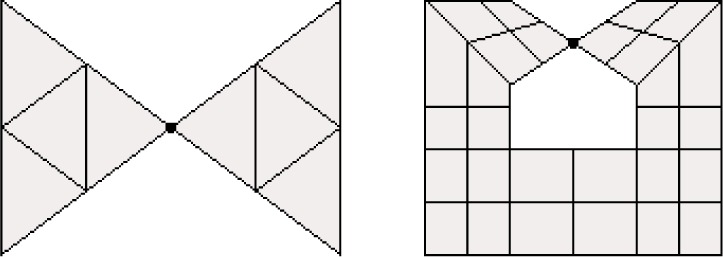
(a) A grid containing a cut vertex. (b) A grid containing a local cut vertex. In each case, the vertex is shown as a large dot.

A vertex, *v*, is called a *cut vertex* if *G* \ *v* is disconnected. See Fig. 2. A *local cut vertex* is a vertex whose removal causes *G* to become disconnected locally. Formally, *v* is a local cut vertex if there exists an *R* > 0 such that for all 0 < ∈ < *R*, (*G* ∩ *B* (*v*, ∈)) \ *v* is disconnected, where *B* (*v*, ∈) is the ball of radius ∈ centered at *v*. Note that a cut vertex is also a local cut vertex. Figure 2(b) illustrates a local cut vertex that is not a cut vertex. In three dimensions, the terms *cut edge* and *local cut edge* are defined similarly.

## 3. Triangles and Tetrahedra

In this section we present what is known about the existence of the different types of Hamiltonian paths and cycles for conforming triangular and tetrahedral grids. We begin with a review of previously published results. We then give the main results of this paper, which are the existence of through-vertex Hamiltonian cycles under very mild conditions. Counterexamples are presented throughout to show conditions under which Hamiltonian cycles and paths do not exist, and to show that the conditions in the hypotheses of the theorems are essential.

Note that the existence of a cycle implies the existence of a path. Therefore most of the existence statements are made for cycles, and it is understood that the same statement holds for paths.

Conversely, non-existence statements are usually made about paths, and it is understood that the same statement holds for cycles. The exceptions to this are when quoting statements from other papers and when a path exists but a cycle does not.

The following theorem is due to Heber et al. [5].

**Theorem 1** *There exists a Hamiltonian path for any conforming triangular grid that contains no local cut vertices*.

The statement of the theorem in Ref. [5] does not mention the absence of local cut vertices or that the grid must be conforming, however the definition of a grid in that paper is that it be a “simplicial complex coming from the simplicial decomposition of a connected 2D manifold” which implies these conditions. The theorem also holds for Hamiltonian cycles, simply by starting the base case in Heber’s inductive proof with a Hamiltonian cycle between two triangles. The hypotheses of the theorem need to be extended slightly because the definition of a cycle assumes at least two elements.

**Corollary 1** *There exists a Hamiltonian cycle for any conforming triangular grid, of size at least 2, that contains no local cut vertices*.

Hamiltonian paths and cycles also exist for tetrahedral grids under similar conditions. This follows immediately from Theorem 2 later in this section.

The following counterexamples show that the absence of cut vertices is an essential condition for Theorem 1, and the absence of local cut vertices is an essential condition for Corollary 1. However, it is not known whether the absence of local cut vertices is essential for the Hamiltonian path.

**Fig. 3:**
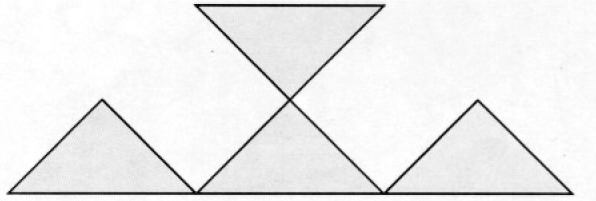
Example of a triangular grid containing cut vertices for which there is no Hamiltonian path.

**Counterexample 1** *There exists a triangular grid containing cut vertices for which there is no Hamiltonian path. See* Fig. 3.

**Fig. 4:**
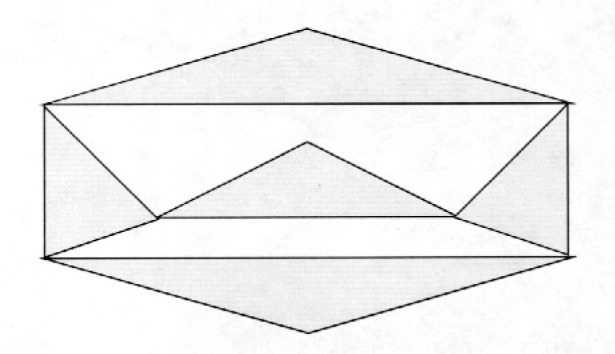
Example of a triangular grid containing local cut vertices for which there is no Hamiltonian cycle.

**Counterexample 2** *There exists a triangular grid containing local cut vertices (but no cut vertices) for which there is no Hamiltonian cycle. See* Fig. 4.

Corollary 1 says that, under very mild conditions, we can always find a Hamiltonian cycle (and hence a Hamiltonian path) in a triangular grid. This is an unconstrained Hamiltonian cycle, i.e., it does not say whether the passage from one element to the next is through a vertex or edge. Indeed, the recursive algorithm implied by the proof of Theorem 1 in Ref. [5] will usually result in passages through both vertices and edges. The obvious question is whether or not through-vertex Hamiltonian cycles or paths and through-edge Hamiltonian cycles or paths exist. The following well-known counterexample shows that we cannot expect to find a through-edge Hamiltonian path in a triangular grid. In fact, determining whether or not a through-edge Hamiltonian cycle exists in a triangular grid is known to be NP-complete [6]. A similar counterexample can be constructed for through-face Hamiltonian paths in tetrahedral grids.

**Fig. 5:**
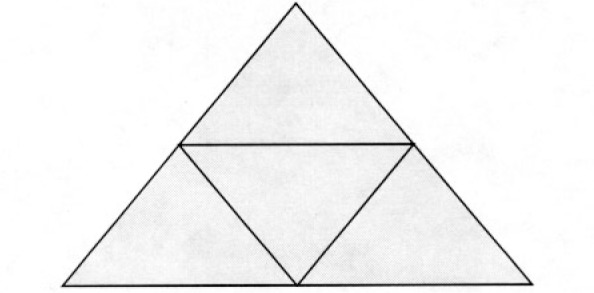
Example of a conforming triangular grid without local cut vertices for which there is no through-edge Hamiltonian path.

**Counterexample 3** *There exists a conforming triangular grid with no local cut vertices for which there is no through-edge Hamiltonian path. See* Fig. 5.

The situation is less grim for through-vertex Hamiltonian cycles. The main result of this paper is that through-vertex Hamiltonian cycles exist for triangular and tetrahedral grids under conditions similar to those for the existence of an unconstrained Hamiltonian cycle. They again require a conforming grid with no local cut vertices. Triangular grids also require that there be at least one interior vertex, and tetrahedral grids also require that there be no local cut edges.

The following lemma says that, under conditions that will arise in the proofs of the main theorems, we can always find two triangles that share a edge, or two tetrahedra that share a face.

**Lemma 1** *Let G be a conforming triangular (tetrahedral) grid with* |*G*| ≥ 2, *no local cut vertices and no local cut edges. Let G*_1_ ⊂ *G contain no local cut vertices and no local cut edges, G*_2_ *= G* \ *G*_1_, |*G*_1_| ≥ 1, *and* |*G*_2_| ≥ 1. *Then*

1. *there exists E*_1_, *E*_2_ ∈ *G such that E*_1_ ∩ *E*_2_ *is a common edge (face), and*
2. *there exists E*_1_ ∈ *G*_1_ *and E*_2_ ∈ *G*_2_ *such that E*_1_ ∩ *E*_2_ *is a common edge (face)*.

**Proof:** For part 1, suppose there are no elements that share an edge (face). Then all connections between elements are vertices (or edges), which must then be local cut vertices (or local cut edges) contradicting the hypothesis that there are no local cut vertices or local cut edges.

For part 2, suppose there is no element in *G*_2_ that shares an edge (face) with an element in *G*_1_. Then *G*_1_ and *G*_2_ are connected by only vertices (and edges), which must then be local cut vertices (or local cut edges). □

We first give the main result for tetrahedral grids, where the proof is shorter.

**Theorem 2** *Let G be a conforming tetrahedral grid with* |*G*| ≥ 2. *If G contains no local cut vertices and no local cut edges then there exists a through-vertex Hamiltonian cycle for G*.

**Proof:** We prove this by induction on |*G*_1_| where *G*_1_ ⊆ *G*, *G*_1_ satisfies the hypotheses of the theorem, and we can exhibit a through-vertex Hamiltonian cycle for *G*_1_.

For |*G*_1_| = 2, let *G*_1_ = {*E*_1_, *E*_2_} where *E*_1_ and *E*_2_ are any two elements that share a common face. Lemma 1 insures the existence of these elements. Let *v*_1_ and v_2_ be two of the vertices that they share. Then *E*_1_*v*_1_*E*_2_*v*_2_*E*_1_ is a through-vertex Hamiltonian cycle for this subgrid.

By induction, let *k* = |*G*_1_| and suppose we have *G*_1_ ⊂ *G*, *k* ≥ 2, *G*_1_ satisfies the hypotheses of the theorem, and *E*_1_*v*_1_*E*_2_*v*_2_…*E_k_v_k_E*_1_ is a through-vertex Hamiltonian cycle for *G*_1_. The grid and cycle can be extended to size *k* + 1 as follows.

Let *E_k_*_+ 1_ ∈ *G* \ *G*_1_ be an element that shares a face with some element *E_i_* ∈ *G*_1_. The existence of *E_k_*_+ 1_ is guaranteed by Lemma 1. Without loss of generality, assume *E_i_* is not *E*_1_ (otherwise, just start the numbering of the cycle with a different element). Since *E_i_* has only four vertices, one of the three vertices shared by *E_i_* and *E_k_*_+ 1_ must be either *E_i_*’s in-vertex *v_i_*_− 1_ or *E_i_*’s out-vertex *v_i_*. Without loss of generality, suppose it is *v_i_* (otherwise, just reverse the ordering of the cycle). One of the three shared vertices, say *v*, must not be *v*_i − 1_ or *v_i_*. Then a through-vertex Hamiltonian cycle in *G*_1_ ∪ *E_k_*_+ 1_ is *E*_1_…*v_i_*_− 1_ *E_i_vE_k_*_+ 1_*v_i_E_i_*_+ 1_…*E*_1_. □

The proof for triangular grids is also a constructive proof that begins with a cycle through two elements and inductively extends this cycle to the complete grid. However, it is a more complicated proof because, in the notation of Theorem 2, we cannot guarantee that *E_k_*_+ 1_ shares a vertex with *E_i_* that is not *v_i_*_− 1_ or *v_i_*. In that case, a more complicated extension of the cycle must be performed.

**Theorem 3** *Let G be a conforming triangular grid with* |*G*| ≥ 2. *If G contains no local cut vertices and has at least one interior vertex then there exists a through-vertex Hamiltonian cycle for G*.

**Proof:** As in Theorem 2, we prove this by induction on |*G*_1_|, and the base case with two triangles is trivial. By induction, suppose we have *G*_1_ ⊂ *G*, |*G*_1_| = *k* ≥ 2, *G*_1_ contains no local cut vertices, and *E*_1_*v*_1_*E*_2_*v*_2_…*E_k_v_k_E*_1_ is a through-vertex Hamiltonian cycle for *G*_1_. The grid and cycle can be extended to a larger size as follows.

Let *E_k_*_+ 1_ ∈ *G* \ *G*_1_ be an element that shares an edge with some element *E_i_* ∈ *G*_1_. The existence of *E_k_*_+ 1_ is guaranteed by Lemma 1. Without loss of generality, assume *E_i_* is not *E*_1_ (otherwise, just start the numbering of the cycle with a different element). One of the two vertices that *E_k_*_+ 1_ shares with *E_i_* must be either *E_i_*’s in-vertex, *v_i_*_− 1_, or *E_i_*’s out-vertex, *v_i_*. Without loss of generality, assume it shares the out-vertex (otherwise, just reverse the order of the cycle). There are four cases to consider.

Case 1. *E_k_*_+ 1_ does not contain *v_i_*_− 1_.

**Fig. 6:**
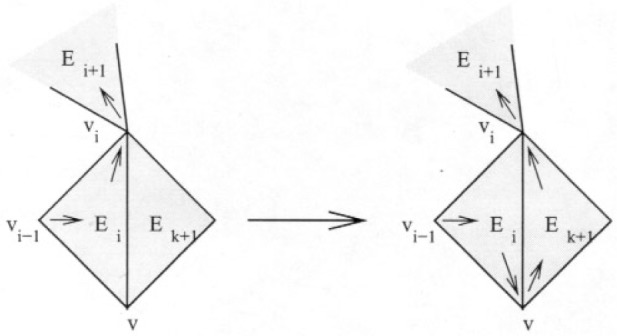
Extension of the cycle for case 1 in Theorem 3.

This is the easy case that corresponds to the proof of Theorem 2. Let *v* be the other vertex shared by *E_k_*_+ 1_ and *E_i_*. Then the new cycle is *E*_1_…*v_i_*_− 1_*E_i_vE_k_*_+ 1_*v_i_E_i_*_+ 1_…*E*_1_. This extension is illustrated in Fig. 6. The arrows pointing from a vertex to the interior of a triangle or from the interior of a triangle to a vertex denote the in-vertex and out-vertex, respectively.

Case 2. *E_k_*_+ 1_ contains *v_i_*_− 1_ and there is another triangle, *E_k_*_+ 2_, not on the current cycle, that shares an edge and *v_i_* with *E_k_*_+ 1_.

**Fig. 7:**
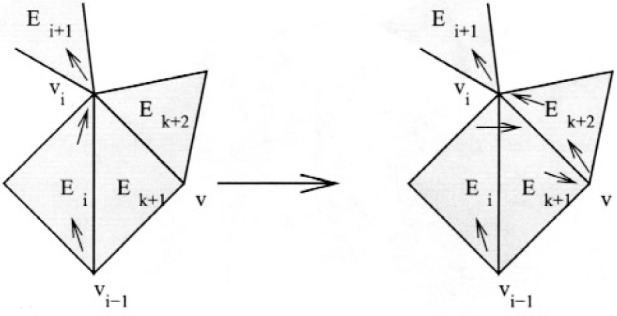
Extension of the cycle for case 2 in Theorem 3.

This case is illustrated in Fig. 7. Let *v* be the other vertex shared by *E_k_*_+ 1_ and *E_k_*_+ 2_. Then the new cycle is *E*_1_…*v_i_*_− 1_*E_i_v_i_E_k_*_+ 1_*vE_k_*_+ 2_*v_i_E_i_*_+ 1_…*E*_1_.

Case 3. *E_k_*_+ 1_ contains *v_i_*_− 1_ and there is another triangle, *E_k_*_+ 2_, that is on the current cycle and shares an edge and *v_i_* with *E_k_*_+ 1_.

**Fig. 8:**
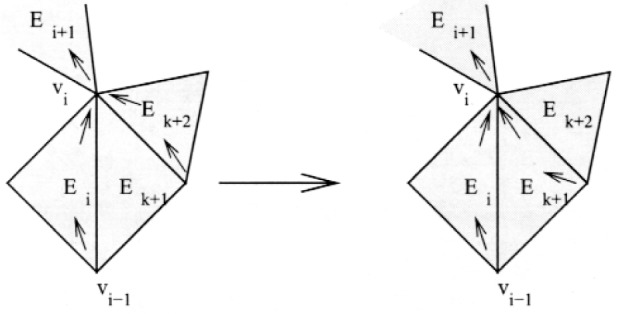
Swapping element *E_k_*_+ 1_ for element *E_k_*_+ 2_ in the cycle.

First note that *E_k_*_+ 1_ must contain both the in-vertex and out-vertex of *E_k_*_+ 2_, otherwise we could apply case 1 with *E_k_*_+ 2_ as *E_i_* to add *E_k_*_+ 1_ to the cycle. Also note that whenever a triangle not on the cycle contains both the in-vertex and out-vertex of a triangle that is on the cycle, we can “swap” this triangle with the other triangle by removing the other triangle from the cycle and inserting the new triangle in its place, as illustrated in Fig. 8.

Case 3a. *v_i_* is an interior vertex.

Swap elements around *v_i_* until either

1. there are two adjacent elements that are not in the cycle (then apply case 2), or
2. there are adjacent elements, one on the cycle and one not on the cycle, where the one not on the cycle does not contain both the in-vertex and out-vertex of the one on the cycle (then apply case 1).

**Fig. 9:**
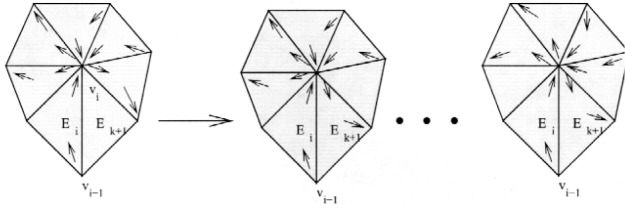
A case where swapping around an interior vertex *v_i_* continues until the other triangle adjacent to *E_i_* is reached.

Note that if we do not encounter two adjacent elements that are not in the cycle (the first subcase), then we must eventually reach the second subcase because the other triangle adjacent to *E_i_* cannot contain the in-vertex of *E_i_*, *v_i_*_− 1_. See Fig. 9.

Case 3b. *v_i_* is a boundary vertex.

**Fig. 10:**
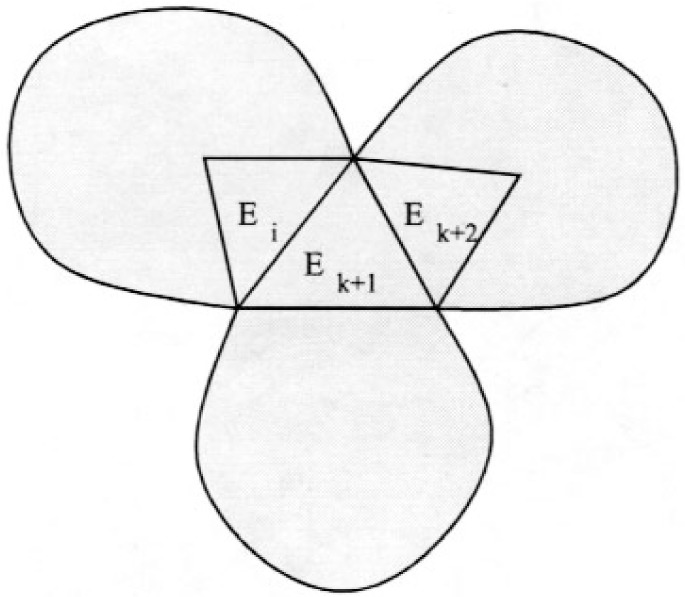
Decomposition of *G* into three components plus *E_k_*_+ 1_ when all three vertices of *E_k_*_+ 1_ are on the boundary.

This implies that all three vertices of *E_k_*_+ 1_ are on the boundary, for if not we could select a different *E_i_*, *v_i_*_− 1_ and *v_i_* (possibly reversing the order of the cycle) such that an interior vertex of *E_k_*_+ 1_ is *v_i_*. This leads to a natural decomposition of *G* into three components plus *E_k_*_+ 1_ as illustrated in Fig. 10. The intersection of any two components is a vertex of *E_k_*_+ 1_. Each component contains a triangle that shares an edge with *E_k_*_+ 1_ because there are no local cut vertices. One component contains *E_i_* and a second component contains *E_k_*_+ 2_. If the third component is not empty, let *E* be the element that shares an edge with *E_k_*_+ 1_. If *E* is not on the cycle, then apply case 2 (reversing the order of the cycle if necessary). If *E* is on the cycle and *E_k_*_+ 1_ does not contain both the in-vertex and out-vertex of *E*, then apply case 1 with *E* as *E_i_*. Thus we can assume that *E_k_*_+ 1_ contains the in-vertex and out-vertex of all adjacent triangles, so it can be swapped with any of them.

**Fig. 11:**
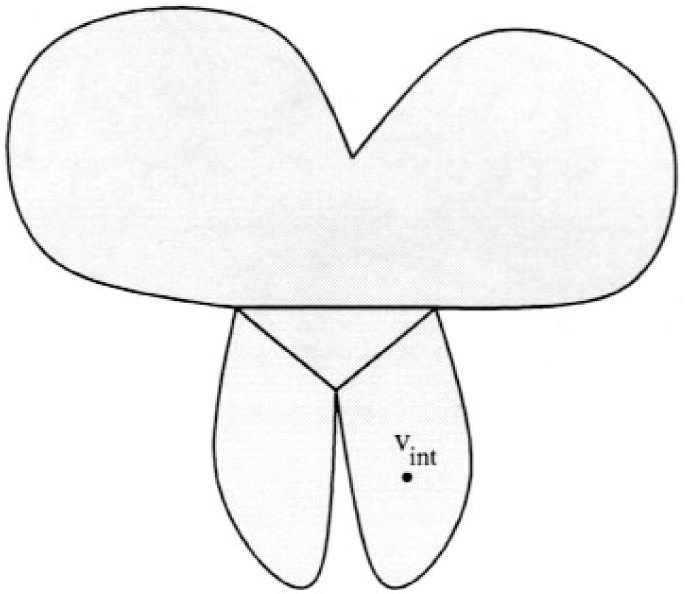
Second decomposition of G into three components plus a triangle after swapping *E_k_*_+ 1_ with a neighbor.

Pick an interior vertex of *G*, *v_int_*. Swap *E_k_*_+ 1_ with the neighbor that is in the same component as *v_int_*. Apply case 1, 2 or 3a to reinsert the other element, if appropriate. If not, then consider the decomposition around the new element and repeat the process (see Fig. 11). The component that contains *v_int_* has at least lost the swapped triangle, and thus is smaller in this decomposition. Since the size of the component containing *v_int_* will continue to shrink with each application of this process, it must eventually lead to *v_int_*, at which point case 3a applies, unless it ends earlier by applying case 1, 2 or 3a.

Case 4. *E_k_*_+ 1_ contains *v_i_*_− 1_ and there is no other triangle that shares an edge and *v_i_* with *E_k_*_+ 1_, i.e., that edge is on the boundary.

Then there must be another triangle that shares *v_i_* and an edge with *E_i_*, for if that edge of *E_i_* was also on the boundary, then *v_i_* would be a local cut vertex. Swap *E_k_*_+ 1_ with *E_i_*, and then apply either case 2 or case 3 to add *E_i_* back into the path. □

The inclusion of an interior vertex is an essential condition for Theorem 3. The same counterexample can be used as was used in Counterexample 3. However, this example does contain a through-vertex Hamiltonian path. It is not known whether or not the inclusion of an interior vertex is essential for the existence of a through-vertex Hamiltonian path.

**Counterexample 4** *There exists a conforming triangular grid with no local cut vertices and no interior vertices for which there is no through-vertex Hamiltonian cycle. See* Fig. 5.

Since the inclusion of an interior vertex “fixes” Counterexample 4, a natural question is whether it can also “fix” Counterexample 3. The following counterexample says that this is not the case.

**Fig. 12:**
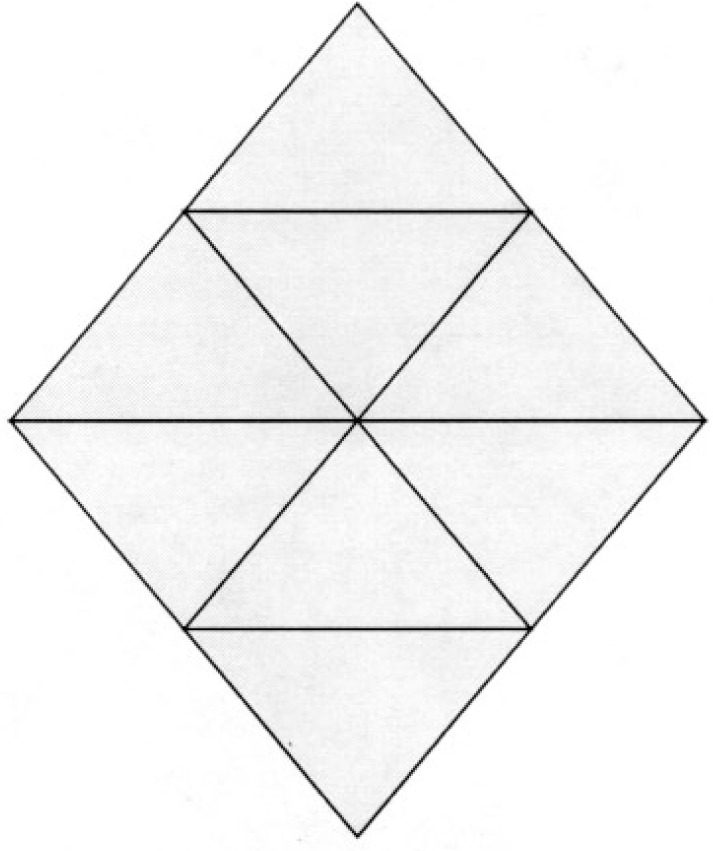
Example of a conforming triangular grid without local cut vertices, but including an interior vertex, for which there is no through-edge Hamiltonian path.

**Counterexample 5** *There exists a conforming triangular grid with no local cut vertices and at least one interior vertex for which there is no through-edge Hamiltonian path. See* Fig. 12.

## 4. Quadrilaterals and Hexahedra

It is not known under what conditions any kind of Hamiltonian path or cycle is guaranteed to exist for quadrilateral and hexahedral grids, or even if there is any characterization. It would certainly be much more stringent conditions than for triangles and tetrahedra, as the following counterexample shows. By replacing the squares with cubes, the same counterexample works for hexahedral grids.

**Fig. 13:**
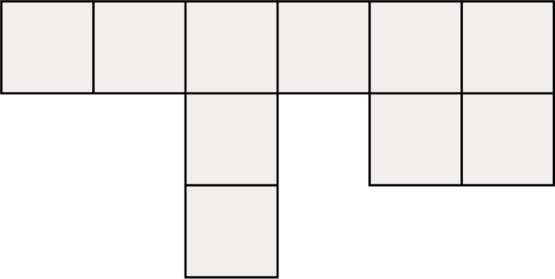
Example of a conforming quadrilateral grid without local cut vertices, but including an interior vertex, for which there is no Hamiltonian path.

**Counterexample 6** *There exists a conforming quadrilateral grid with no local cut vertices and at least one interior vertex for which there is no Hamiltonian path. See* Fig. 13.

The proofs of Theorems 2 and 3 break down when applied to quadrilaterals and hexahedra because, in the notation of those theorems, *E_k_*_+ 1_ is not guaranteed to contain either the in-vertex or out-vertex of *E_i_*. Without that condition, it is more difficult, and in some cases impossible, to modify the cycle in a way that adds *E_k_*_+ 1_ to it.

However, there are some other transformations of the cycle that can be applied to insert *E_k_*_+ 1_ into the cycle in some situations. These include some situations where the grid is 1-nonconforming. Most of these transformations apply not only to quadrilaterals and hexahedra, but to any shape of element, including triangles and tetrahedra, and to mixed elements. Thus one could write a general program that applies to any grid. The illustrations will show the application of the transformations to quadrilaterals. They show examples of the transformations, but are not exhaustive.

An algorithm that attempts to find a through-vertex Hamiltonian cycle begins by picking two elements that share an edge (face in three dimensions) and constructing a cycle consisting of these two elements and two of the vertices they share. Then repeatedly pick an element that is not in the cycle but shares an edge (face in three dimensions) with an element in the cycle, and attempt to add it to the cycle by trying all of the following transformations that apply until one succeeds. If none of them succeed then try another element and come back to this one later. If there are elements not yet added to the cycle and none of the transformations work with any of the remaining elements, then you must insert a “broken link” creating a discontinuity in the path, i.e., insert an element such that either it is not adjacent to the previous or next element in the cycle, or the in-vertex and out-vertex do not match. When all elements have been added to the (possibly broken) cycle, the algorithm finishes.

In the following transformations, the cycle contains the segment *v_i_*_− 2_ *E_i_*_− 1_ *v_i_*_− 1_ *E_i_v_i_E_i_*_+ 1_ *v_i_*_+ 1_ and *E* is an element that shares an edge (face in three dimensions) with *E_i_* and is not in the cycle.

**Fig. 14:**
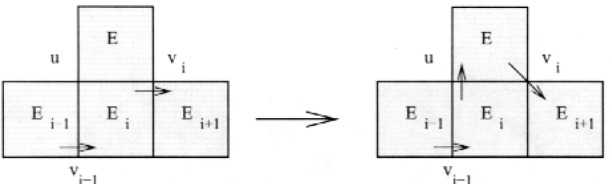
Example of Transformation 1.

**Transformation 1** *If E contains v_i_ and shares another vertex, u ≠ v_i_*_− 1_, *with E_i_, then replace the segment with v_i_*_− 2_*E_i_*_− 1_*v_i_*_− 1_*E_i_uEv_i_E_i +_*_1_*v_i +_*_1_. *See* Fig. 14.

This is the same transformation that was used in the proof of Theorem 2.

**Transformation 2** *If E contains v_i_*_− 1_ *and shares another vertex, u ≠ v_i_, with E_i_, then replace the segment with v_i_*_− 2_*E_i_*_− 1_*v_i_*_− 1_*EuE_i_v_i_E_i +_*_1_*v_i +_*_1_.

This is like the previous transformation, but using the in-vertex instead of the out-vertex.

**Fig. 15:**
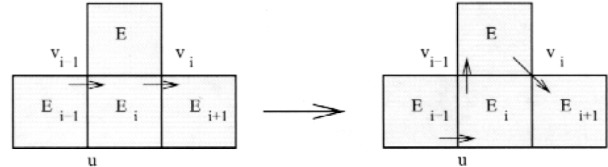
Example of Transformation 3.

**Transformation 3** *If E contains both v_i_*_−_ *_1_ and v_i_, and E_i_*_−_ *_1_ shares another vertex, u ≠ v_i_*_− 2_ *with E_i_, then replace the segment with v_i_*_− 2_*E_i_*_− 1_*uE_i_v_i_*_− 1_*Ev_i_E_i +_*_1_*v_i +_*_1_. *See* Fig. 15.

**Transformation 4** *If E contains both v_i_*_− 1_ *and v_i_, and E_i +_*_1_ *shares another vertex, u ≠ v_i +_*_1_ *with E_i_, then replace the segment with v_i_*_− 2_*E_i_*_− 1_*v_i_*_− 1_*Ev_i_E_i_uE_i +_*_1_*v_i +_*_1_.

This is like the previous transformation, but with the change occurring at the out-vertex of *E_i_* instead of the in-vertex.

**Fig. 16:**
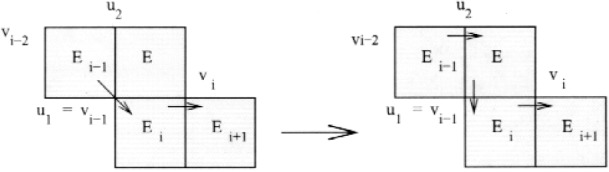
Example of Transformation 5.

**Transformation 5** *If E shares a vertex u*_1_ *≠ v_i_ with E_i_, and shares a vertex u*_2_ *with E_i_*_− 1_, *u*_2_ *≠ u*_1_ *and u*_2_ *≠ v_i_*_− 2_, *then replace the segment with v_i_*_− 2_*E_i_*_− 1_*u*_2_*Eu*_1_*E_i_v_i_E_i +_*_1_*v_i +_*_1_. *See* Fig. 16.

**Fig. 17:**
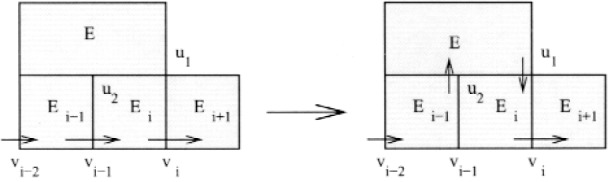
Example of Transformation 5 with hanging node.

This transformation can also handle some forms of hanging nodes, as shown in Fig. 17.

**Transformation 6** *If E shares a vertex u*_1_ *≠ v_i_*_− 1_ *with E_i_, and shares a vertex u*_2_ *with E_i+_*_1_, *u*_2_ *≠ u*_1_ *and u*_2_ *≠ v_i +_*_1_, *then replace the segment with v_i_*_− 2_*E_i_*_− 1_*v_i_*_− 1_*E_i_u*_1_*Eu*_2_*E_i +_*_1_*v_i +_*_1_.

This is like the previous transformation, but with the changes occurring at *E_i_*_+ 1_ instead of *E_i_*_− 1_.

**Fig. 18:**
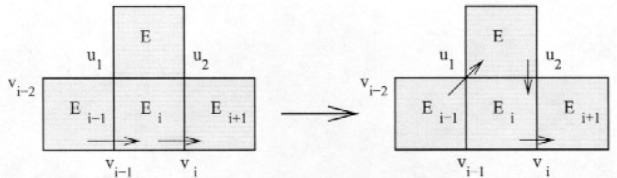
Example of Transformation 7.

**Transformation 7** *If E contains neither v_i_*_− 1_ *nor v_i_, but it shares a vertex u*_1_ *with E_i_*_− 1_, *u*_1_ *≠ v_i_*_− 2_, *and shares a vertex u*_2_ *with E_i_, u*_2_ *≠ u*_1_, *then replace the segment with v_i_*_− 2_ *E_i_*_− 1_*u*_1_*Eu*_2_*E_i_v_i_E_i +_*_1_*v_i +_*_1_. *See* Fig. 18.

**Transformation 8** *If E contains neither v_i_*_− 1_ *nor v_i_, but it shares a vertex u*_1_ *with E_i +_*_1_, *u*_1_ *≠ v_i +_*_1_, *and shares a vertex u*_2_ *with E_i_, u*_2_ *≠ u*_1_, *then replace the segment with v_i_*_− 2_*E_i_*_− 1_*v_i_*_− 1_*E_i_u*_2_*Eu*_1_*E_i +_*_1_*v_i +_*_1_.

This is like the previous transformation, but with the changes occurring at *E_i_*_+ 1_ instead of *E_i_*_− 1_.

**Transformation 9** *If E contains v_i_ and there is another element, F, that is not on the cycle, shares a vertex, u*_1_ *≠ v_i_, with E, and shares a vertex u*_2_ *with E_i_, u*_2_ *≠ u*_1_ *and u*_2_ *≠ v_i_*_− 1_, *then replace the segment with v_i_*_− 2_*E_i_*_− 1_*v_i_*_− 1_*E_i_u*_2_*Fu*_1_*Ev_i_E_i +_*_1_*v_i +_*_1_.

**Fig. 19:**
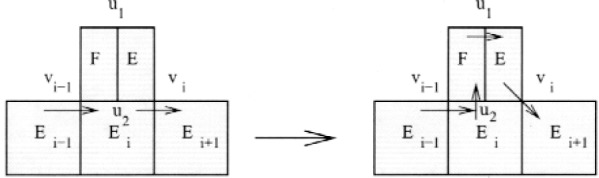
Example of Transformation 9.

This transformation can handle some instances of hanging nodes, as shown in Fig. 19. It can also handle case 2 in the proof of Theorem 3, although it places the elements in a different order than that used in the proof.

**Transformation 10** *If E contains v_i_*_− 1_ *and there is another element, F, that is not on the cycle, shares a vertex, u*_1_ *≠ v_i_*_− 1_, *with E, and shares a vertex u*_2_ *with E_i_, u*_2_ *≠ u*_1_ *and u*_2_ *≠ v_i_, then replace the segment with v_i_*_− 2_*E_i_*_− 1_*v_i_*_− 1_*Eu*_1_*Fu*_2_*E_i_v_i_E_i +_*_1_*v_i +_*_1_.

This is like the previous transformation, but with *E* sharing the in-vertex of *E_i_* instead of the out-vertex.

**Transformation 11** *If E contains both v_i_*_− 1_ *and v_i_, then replace the segment with v_i_*_− 2_*E_i_*_− 1_*v_i_*_− 1_*Ev_i_E_i +_*_1_*v_i +_*_1_.

This is the swapping of one element for another that was described in case 3 of the proof of Theorem 3. One would next attempt to add *E_i_* back into the cycle. If done recursively, this will handle case 3b of Theorem 3.

## 5. Conclusion

We considered the existence of various types of Hamiltonian paths and cycles in two-dimensional grids consisting of triangles or quadrilaterals, and three-dimensional grids consisting of tetrahedra or hexahedra. The types of Hamiltonian paths and cycles are distinguished by whether passage from one element to the next must be associated with an edge (through-edge Hamiltonian), must be associated with a vertex (through-vertex Hamiltonian), or can pass through either (unconstrained Hamiltonian). The existence results presented in this paper can be summarized as:

1. There exists an unconstrained Hamiltonian path in any conforming triangular grid with no local cut vertices (previously known result). This is easily extended to Hamiltonian cycle.
2. There exist triangular grids for which there is no through-edge Hamiltonian path (previously known result). This also holds for through-face Hamiltonian paths in tetrahedral grids.
3. There exists a through-vertex Hamiltonian cycle (and hence through-vertex Hamiltonian path) for any conforming triangular grid with no local cut vertices and at least one interior vertex.
4. There exists a through-vertex Hamiltonian cycle (and hence through-vertex Hamiltonian path, unconstrained Hamiltonian cycle and unconstrained Hamiltonian path) for any tetrahedral grid that contains no local cut vertices and no local cut edges.
5. There exist rectangular grids for which there is no Hamiltonian path or cycle of any type. This also holds for hexahedral grids.

Examples were given to show that the stated conditions are essential. Some open questions remain:

1. It is not known if the absence of local cut vertices is essential for the existence of a Hamiltonian path in a triangular grid. (The absence of cut vertices is essential, and the absence of local cut vertices is essential for a Hamiltonian cycle.)
2. It is not known if the presence of an interior vertex is essential for the existence of a Hamiltonian path in a triangular grid. (It is essential for a Hamiltonian cycle.)
3. The conditions under which any type of Hamiltonian path or cycle exists in a rectangular or hexahedral grid are not known.

The existence proofs for through-vertex Hamiltonian cycles in triangular and tetrahedral grids are constructive proofs. An efficient algorithm to find such a cycle can be derived from the construction in the proofs. Another algorithm, which can be applied to elements of any shape, was outlined. This algorithm is not guaranteed to find a through-vertex Hamiltonian cycle even if one exists, but it is likely to produce a (possibly broken) cycle that is close.
